# Publicness and commoning: Pandemic intersections and collective visions at times of crisis

**DOI:** 10.1177/13678779211060363

**Published:** 2022-07

**Authors:** Myria Georgiou, Gavan Titley

**Affiliations:** 4905 London School of Economics and Political Science – LSE, UK; 8798 Maynooth University, Ireland

**Keywords:** collective vision, commons, commoning, Covid-19, crisis, pandemic, politics of solidarity, publicness, public sphere

## Abstract

In this article, we examine *publicness* during the pandemic, with a particular focus on the conditions it creates or constricts for engagement, solidarity and collective action. We interrogate the intensive publicness of the crisis to reflect on its assumed and established equation with progressive political possibility – transparency, accountability and democratic procedure. Theoretically, we cut into the contemporary ambiguity of publicness by putting it into conceptual dialogue with the idea of *commoning*, a notion that speaks to the resources and political consequences of coming together, and publicness not as coexistence and speech acts but as a domain of struggle. By considering the intersection of publicness and commoning, we aim to provide one way of thinking about how and when public revelation can be oriented towards material and political change. We propose three lines of examination: *publicness without commoning*; *publicness with contingent commoning*; and *commoning without publicness*.

## Introduction: pandemic as a crisis of publicness?

The interlocking social, economic and political crises precipitated and deepened by the Covid-19 pandemic have engendered a pronounced sense that while another world may be possible, above all, it is necessary. The unprecedented and enforced economic slowdown since spring 2020 has, almost since its inception, been narrated as a moment of radical ecological opportunity, with the euphoric ephemera of dolphins ‘re-appearing’ in the canals of Venice giving way to sustained claims that this period may represent a ‘major turning point in world history’ if the conjunctural disruption can fuel momentum for concerted action on climate change (Caradonna, 2021). The stalling of economic growth, and the slowing and jamming of international trade and mobility, has exposed the contingency and reversibility of capitalist globalisation, raising questions as to its supposedly unquestionable systemic resilience. The frequently hurried governmental re-discovery of massive public subsidy and investment, and the – often reluctant – prerogative of support for key industries and emergency welfare programmes, has further eroded neoliberalism's frayed mantra that ‘there is no alternative’. The pandemic relentlessly demonstrated the extent of labour inequalities, reinvigorating debates about the future of work, and posing questions about the classed and gendered dimensions of ‘working from home’. The coincidence of the pandemic's grossly disproportionate impact on racialised minorities with the transnational anti-racist mobilisation of Black Lives Matter following the murder of George Floyd – not to mention the widespread racialising of the pandemic's causes and ‘carriers’ (Clergé and Edwards, 2021) – has empowered sustained mobilisation against the racialised violence of states and the continuities and legacies of the colonial system in the present.

These headline conjectures are not intended to minimise the extraordinary suffering produced by the pandemic, nor to elide the patterned inequities in its distribution. Nor are they presented, simplistically, as openings or opportunities, as each of these unsettled dimensions is also the site of burgeoning reaction and retrenchment, from the recently observed turn to ‘authoritarian capitalism’ (Macfarlane, 2020; Meadway, 2021) and the transnational articulation of an equally authoritarian ‘anti-anti-racism’ (Titley and Lentin, 2021). And, of course, such initial observations require swift and careful contextual delineation to have any sustainable critical value. Nevertheless, they serve as indicators of the ways in which a widespread sense of profound disruption is animating public debate, arguably producing, at minimum, a heightened realisation of the interconnected nature of contemporary problems. Increasingly, reflective accounts note how this in turn produces the conditions for hope and optimism, where popular sensibilities can attune to the need for profound ecological, political-economic, social transformation. As Jonathan Gross argues, ‘Covid-19 has derailed hegemonic accounts of the future, making it possible and necessary to re-imagine many dimensions of human life’ (Gross, 2021: 2). Yet, as the pandemic moves into a stage of more diffuse and unequal impacts across and within states, Gross’s follow-up questions are pressing – ‘But who will do the imagining? Who will write the scripts of hope? And where exactly will this writing happen?’ (Gross, 2021: 2).

One key site of this re-imagining and scripting is in public, or the public sphere, and the sequence of statement and question in Gross’s account underlines two dimensions of relevance to publicness as a resilient democratic imaginary. The first is that the intensive dislocations of the pandemic period are frequently regarded as constituting a process of revelation, of rendering transparent and exposing the nature of *the problem*. Second, this horizon of publicness, the condition of having been rendered public, is further assumed as mobilising the basis for some form of collective response. In *The Revenge of the Real: Politics for a Post-pandemic World*, Bratton (2021: loc. 121) captures this relation overtly: ‘instead of naming this moment a “state of exception”, we should see it more as revealing pre-existing conditions.… We must keep attention trained on the pathologies revealed, and in so doing wilfully inhabit a changed world and its many challenges.’

And yet this insistent idea that the pandemic has exposed and revealed problems in public, and that this provides the basis for a public to respond, is articulated under conditions that are often regarded as constituting a crisis of publicness. The widespread media attention paid to the proliferation of Covid-origin and ‘anti-vaxx’ conspiracy theories is an obvious dimension of this, but these conditions clearly pre-date the pandemic. While powerful traditions of normative thought have sought to delineate the processes and procedures conducive to public understanding and democratic action (see Ferree et al., 2002), media sociology has increasingly focused on the significance of conditions of media and social fragmentation (Andrejevic, 2013; Keane, 2013), amplified political dissonance (Pfetsch, 2018), and communicative and informational abundance (Citton, 2017) on the constitution of public knowledge and understanding. Research on ‘post-democracy’ and the diminished relations between popular participation and representative democracy has questioned the assumed relation between – vastly increased and intensified – public communication and political agency in what Jodi Dean has termed ‘democracies that speak without listening’ (Dean, 2009; see also Fenton and Titley, 2015; and on post-democracy Crouch, 2004; Mair, 2013). The global pandemic can also be regarded as exacerbating these conditions. Beyond the specific and spectacular focus on conspiracy theories (for a discussion see Guilhot, 2021), significant anxieties over politically motivated disinformation and the sheer volume and velocity of informational production have accentuated the constitutive uncertainty of information associated with ‘risk society’ and ‘post-truth’ paradigms. This informational instability has underlined another dimension of the powerful publicness of the crisis, as it unfurls under conditions of intense mediation, incessant public relations, and polarised political communication, and an uneven if undeniable recognition of how the pandemic has exacerbated existing inequalities.

The problem we seek to explore, therefore, is the following. There exists a palpable critical hope that the pandemic has ‘revealed’ conditions that generate public awareness, and thus the possibility of concerted political action. This awareness, at the same time, must emerge from the complex and uncertain conditions of contemporary publicness. The publicness of the pandemic's impacts renders palpable the need for more thinking and working together, while the dynamics of publicness render the production of collective understanding and mobilisation more fractious and difficult. For this reason, we interrogate publicness during the pandemic, with a particular focus on the conditions it creates or constricts for engagement, solidarity, and collective action. We examine the intensive publicness of the crisis to reflect on its assumed and established equation with progressive political possibility – transparency, accountability and democratic procedure. The informational dynamics of the pandemic have rendered this a significant area of current commentary and research (Bratton, 2021; Gross, 2021; Nguyen et al., 2020, among others), and thus our contribution to this discussion is limited and specific. Theoretically, we cut into the contemporary ambiguity of publicness by putting it into conceptual dialogue with the idea of *commoning*.

Why commoning? The political hope vested in what the pandemic reveals about current inequalities and injustices is oriented to creating the conditions for their amelioration or abolition, through the mobilisation of some form of collective understanding and action. The idea of the commons has been widely invoked in this regard, as we discuss subsequently in relation to vaccine nationalism. More importantly, it is a notion that encompasses both resources and relations, end goals and the processes of cooperation and solidarity required to secure and sustain them (Caffentzis and Federici, 2014; Harvey, 2011). These resources and relations must be cultivated and mobilised under conditions of complex publicness, and this publicness must be navigated as an ambivalent dimension of commoning. By considering their intersection, we aim to provide one way of thinking about how and when public revelation can be oriented towards material and political change. We propose three lines of examination.

The first, *publicness without commoning*, examines the enormous, transnational public debate about vaccine access and justice, and the impotence of publicness in the face of both vested interests and a democratically unaccountable intellectual property regime. The second, *publicness with contingent commoning*, examines the political struggle to frame the pandemic as a public health crisis requiring collective understandings and orientations, and considers the question of such political imaginaries under conditions of unevenly distributed social isolation and political fragmentation. The third, *commoning without publicness*, confronts the value attributed to revelation and transparency by discussing forms of solidarity and dissent that, because they are anti-systemic, outside the acceptable norms of publicness or at the social margins, have either been expelled from the public domain, or have sustained themselves by eluding surveillance and punitive publicity. While these three lines of examination generate useful conceptual problems, they are primarily chosen because of their palpable relevance as intersections that have been forcefully articulated during the pandemic, and at different scales of agency – the international system, the ‘national society’, and the urban margins subject to oscillating dynamics of invisibility and hyper-visibility. As noted, the unequal impacts of the pandemic within and across nations pose a challenge for abstract argument, and for that reason the examples used are drawn from our respective contexts of the United Kingdom and the Republic of Ireland.

## Publicness

The unprecedented nature and scale of the pandemic, the severity of its social and economic impacts, and the extent of the – unequally distributed – demands and responsibilities it placed on ordinary people required, from the beginning, overt recourse to the public as a democratic, but also social, imaginary. Consider a heuristic example. In a special televised address to the nation on St Patrick's Day, 17 March 2020, the then-Taoiseach (prime minister) of Ireland Leo Varadkar outlined the first governmental responses to the coming Covid-19 pandemic, and exhorted his audience to do their bit too, inviting them to ‘come together as a nation by staying apart’. His comment was immediately excerpted in headlines and on Twitter as a highlight of the speech. The rhetorical paradox captures something of the lived paradox of ‘social distancing’ in a public health emergency, where a practice usually associated with stigmatisation and marking out ‘deviance’ is flipped and re-valorised as a basic dimension of communal interaction (Nerlich and Jaspal, 2021). To broach this abrupt re-coding of social norms, Varadkar invokes the public in highly familiar ways, as a ‘kind of social totality’ (Warner, 2002: 49) primed to listen and act, and as an imagined community already possessed of the orientations, values and resources required to triumph in the face of adversity. It is this imaginary of a coherent totality that anchors the unarticulated power of publicness as a process of revelation and consequence; the pandemic can be explained, and the public will understand, and consequently act collectively.

Initial sociological accounts in Ireland and elsewhere in spring 2020 attested to the powerful performativity of collective invocations of the public under conditions of anxiety and uncertainty – an interesting measure of this is the significantly renewed engagement reported by public service broadcasters with their live news events (Snoddy, 2020). However, this kind of performative projection, always partial in its reach and reception, rapidly frayed on contact with the unequal impacts of the pandemic being rendered public. Varadkar's invitation to come together, addressed to a specifically national community, excluded from the start asylum-seekers warehoused by the Irish state in a system of ‘direct provision’ which, like other residential settings, immediately proved particularly vulnerable to the spread of the virus. More broadly, the elite political appeal to a resilient national collective is greeted, following the financial crisis and austerity politics of 2011 onwards, with widespread suspicion in Ireland (Coulter, 2015). Thus, a significant line of political critique and opposition during this period pivoted on juxtaposing this rhetorical vision of the public with the socio-economic realities publicly revealed by the pandemic. The sociologist Ryan Nolan, writing on the exacerbation of social inequality in Ireland during the pandemic, summarises this mode of contention:

The Covid-19 pandemic has illuminated the stratification of society in every nation-state it has touched. The pandemic has unmasked the hidden systems of inequality that are lost in the mundanity of everyday life. The veneer of capitalist meritocratic society has been fractured. The disruption of social order has ‘breached’ the social world publicising structures of inequality previously obscured.  (Nolan, 2021: 45)

Illumination, unmasking, fractured veneers, and publicness – the role and value of the public in representative democracies is evaluated very differently across political traditions and tendencies, but some form of critical faith in the mobilising power of public revelation is widely distributed. As Ferree et al. (2002) note, in their useful typology of theories of the public sphere, an emphasis on transparency as the key democratic value of publicness is often a feature of ‘elite dominance’ in ‘representative liberalism’, as it grounds the legitimacy of expert leadership, institutional trust and party-dominance in ‘well-functioning’ democracies:

From this perspective an important criterion of good public discourse is its transparency. It should reveal what citizens need to know about the workings of their government, the parties that aggregate and represent their interests, and the office-holders they have elected to make policy on their behalf. Inclusion is important, not in the sense of giving ordinary citizens a chance to be heard, but in the sense that representatives should have the time and space to present their contrasting positions fully and accurately. (Ferree et al., 2002: 291)

Within debates about public sphere theory, critiques of these positions have been widely rehearsed (Ferree et al., 2002). However, while these debates focus on processes of inclusion, the character of discourse and the problem of unitary conceptions of ‘the public’, the revelatory power of publicness receives somewhat less attention. If, as Charles Taylor has argued, the idea of publicness as a process of (mutual) appearance is a defining feature of modern social imaginaries (Taylor, 2003), it is given further ballast in two closely related evaluations in political philosophy. The first is a republican insistence on argumentation as the modality which produces publicness through the transcendence of ‘private experience’, and, second, a liberal investment in transparency and attention as checks on (private) power (see Kunelius, 2013: 31–2). A critical materialist position such as Nolan's does not depend on either liberal or republican precepts, rather it configures revelation as a basis for rational commitment to political work.

While broadly sharing this commitment, we suggest that assumed relations between publicness, transparency and collective agency require more consideration in relation to both democracy and communication. Research on the politics of democratic capture and ‘post-democracy’, for example, point to the constitutive de-linking of transparency from democratic procedures. What Dean terms ‘norms of publicity that emphasise transparency and accountability’ (Dean, 2009: 21) – potentially enhanced by the surveillance and reactivity of the digital media ecology – serve to generate media content and debate, but do not produce, for all the intensity of circulation, political consequences. Presaging the focus on ‘post-truth’ that gave expression to democratic anxiety in the Anglophone world during the Trump presidency, Andrejevic’s (2013) discussion of ‘info-glut’ challenges the extent to which received investments in publicness can navigate a changing landscape of information and power. That is, while the classic relation between information and power imagines more publicness as diluting ideological control and revealing vested interests and ideological projects, the sheer abundance and speed of contemporary communications and information dynamics turns publicity back on itself in an endlessly relativising circuitry. In this environment, rendering meaning unstable can be a way of controlling it, highlighting ‘the contingency, indeterminateness, and ultimately the helplessness of so-called truth in the face of power’ (Andrejevic, 2013: 9).

The pandemic has generated increased faith in the orienting power of publicness. It has also been an intensely mediated and divergently experienced temporal event shaped by these types of communicative and informational dynamics. By taking up this faith and ambiguity in relation to ideas of commoning, we seek to think about ways in which transparency and revelation can be connected to collective processes and possibility.

## Commoning

Turning to the idea of commoning allows us, first, to note resilient problems with the established notions of publicness solidified in the context of the pandemic. The legitimacy that publicness accords is unevenly allocated, with certain values and performances of being and acting in public privileged against others which have been pathologised and chastised. Repeatedly, conceptions of national unity, institutional authority and individualised digital connectivity have been projected as acceptable, desired and ‘safe’ forms of publicness against unauthorised and collective efforts to tackle the epidemiological crisis and its unevenly distributed economic and social effects. Yet notions of institutionalised and mediated publicness – combined with the presumed ‘safety’ of domestic and digitally mediated privacy – did little to address the intensified inequalities of the pandemic, with many women isolated within their unsafe homes, migrants detained in the name of public health, and many urban poor forced to navigate scarce access to basic provisions (Alim, 2020; Grierson, 2021). If anything, established and reinforced conceptions of publicness had little space for those unable to fit within a neat public/private divide as well as those demanding and enacting a politics of solidarity and care.

Against a permissible order of public appearance, the concept of the commons is instead concerned with a ‘differentiated publicness’ of conjoined action (Sohn et al., 2015) and the possibility of cooperation and solidarity against domination and exploitation (Caffentzis and Federici, 2014). The commons thus speaks to the political consequences of coming together, and publicness not as reflexive coexistence and mere speech acts but as ‘a struggle against enclosures, against the privatization of spaces of freedom, against exclusion, and perhaps most importantly, against private property’ (Ticktin, 2020).

Conceiving public participation as a process rather than as a static condition formulated through the predetermined roles of its actors, theories of the commons identify the possibilities for forming solidarities and alliances out of shared orientations, visceral needs or collective precarity. These processes are dynamic, contextual, and porous, as for example seen in urban commons’ green projects (Finn, 2014), mobile commons’ migrant solidarities (Trimikliniotis et al., 2015), or feminist commons’ politics of care (Ticktin, 2020), all of which emerge to tackle debilitating inequalities and threats to life and wellbeing. Recognising the possibilities of confronting inequalities and exclusions as they emerge in sites of life, these same debates have increasingly moved towards an emphasis on *commoning* (Harvey, 2011): the open-ended, incomplete and contradictory process of creating spaces – digital and material – for common action. Consequently, commoning has been increasingly associated with radical social epistemologies to address: possibilities of solidarity outside or against controlled and surveilled digital and material spaces; imaginaries of collectivities and communities of choice; configuration of new collective identities around joint action rather than origin and privilege; and acts of anti-systemic and radical redistribution of material resources, such as public space (especially in urban commons) and symbolic resources such as knowledge and digital skills (especially in digital commons).

The vocabulary of the commons is instrumental to theorising publicness as a process (Kavada and Poell, 2021), thus allowing us to see the connection and convergence of different actors not only through discourse but also through practice, and outside the determination of institutions of the state, the media and the market. This implies recognising the power of physical (and digital) bodies assembling through a politics of solidarity (Butler, 2018); transversal assemblages (Braidotti, 2019) challenging the control of public space and its technologies so as to generate various acts of dissent; or civic projects, such as open-source ‘commons-based peer production’ (Bradley, 2015) organised on the basis of ‘the principle of social cooperation and the defence of the already existing forms of communalism’ (Caffetzis and Federici, 2014: 96). Commoning in this context is about struggle as much as mutuality. It is a concept that challenges both notions of homogeneous publics and the privatisation of public space, discourse and technologies for profit, and instead conceives publicness as mobilisation that can make a difference to collective lives (Finn, 2014).

In the times of the pandemic, conceptions and practices of commoning have contested the exacerbated exclusions of national imaginaries, individualised care, and mobility surveillance. Non-hierarchical, multi-scalar and horizontal forms of sociality and capacious infrastructures of political care, Ticktin (2020) argues, enhance experiments with the commons – for her, a feminist commons. Coming together, she argues, shows that ‘paradoxically, rather than isolating, to stay healthy, people are forging new egalitarian forms of connection’. The masked crowds of the anti-racist protests, for example, represent such new forms of connection, with masks protecting against ‘individualized police surveillance’ (Ticktin, 2020), as well as the virus. It also creates possibilities for the emergence of a generic subject ‘joined in political – not personal – relations’, connected through shared anger irrespective of shared identities. Similarly, mutual aid groups emerging in big city neighbourhoods rearticulated a politics of solidarity through practices of commoning (Georgiou, 2020), sharing resources and care responsibilities beyond pre-existing relations and, in the process, counterbalancing state failure to provide sufficient food and medical care.

Thinking about processes and horizons of commoning offers an opening to consider what publicness produces and can produce, beyond revelatory speech acts and institutionalised conceptions of transparency. Nonetheless, acts of commoning, as much as the formulation of commons against exclusions and inequalities, are not to be idealised, as they are inevitably marked by their own contradictions and conflicting claims. Further, disjuncture between the expansive character of publicness and the often restricted possibilities for commoning are politically ambivalent, generating both determination and resignation. To explore this further, we discuss three different ways in which publicness and commoning have intersected contextually, thematically and at varying scales of action during the pandemic to date.

## Publicness without commoning

As early as October 2020, prior to the approval and public licensing of Covid-19 vaccines in the wealthy countries of the ‘West’, the economist David McAdams warned that vaccine politics threatened to precipitate a globalised tragedy of the commons, whereby the rush to procure – and to be seen to procure – vaccine supplies for national constituencies not only damaged the capacity of many nation-states to access them, but also actively obscured the implacably globalised nature of the pandemic threat (Gordon, 2020). That is, for all the breathless discussion of ‘herd immunity’ in many national contexts, functioning global immunity required widespread vaccine access, and this access was being stymied by a complex web of private interests, legal frameworks, governance structures, political inaction and ‘capitalist realism’ (Fisher, 2009).

As Sariola (2021) documents, the architecture of intellectual property rights (IPR) overtly protects ‘industry benefits over human health and well-being’. It may not be surprising that the ‘epidemiological short-sightedness’ of ‘vaccine capitalism’ is impervious to humanitarian or global justice arguments. More unexpected is how this seems to mitigate against the functioning of capitalism as an interconnected global system, privileging the pharmaceutical industry over other major sectors stunted by the slowdown in trade and human flows. As Sariola summarises:

The commonly presented claim that IPRs protect innovator companies from market failure and financial risks do not apply in the case of COVID-19 vaccines because the research was done predominantly on public funding from various governments in the Global North, which means that companies have to invest very little, and there continues to be an enormous market for vaccines. COVID-19 vaccines should be treated as global public goods because at present, the protection of IPRs to the vaccine companies are causing health and socioeconomic suffering globally, rather than alleviating them. (Sariola, 2021)

In the debates and campaigns which have accompanied this protectionism, the impediments to treating vaccines as ‘global public goods’ have been given various labels. In their totality, they set out the mesh of political, economic and legal dimensions which restrict action on intellectual property barriers and thus on the just distribution of a common resource. While ‘vaccine capitalism’ adheres to exclusivist and monopolist imperatives, leavened with charitable donations by wealthy nations of ‘surplus’ stock (Mookim, 2021), the idea of vaccine nationalism specifies the intersection of ‘national interests’ with the systemic dynamics of market provision. Competitive procurement of vaccines and domestic political considerations – the pressure to be seen to ‘defeat’ the virus – has driven the over-accumulation and hoarding of stock even as national and international agencies warn of the critical importance of global vaccination in ‘self-interested’ epidemological terms, that is, radically uneven patterns of immunity risk accelerating the evolution of ‘escape variants’ that weaken the efficacy of the vaccines administered and hoarded in wealthy nations (Katz et al., 2021; Lagman, 2021). Ideas of vaccine imperialism (Vanni, 2021), colonialism (Olla, 2021) and apartheid (Shabi, 2021) situate hoarding practices and profit imperatives in the *longue durée* of globalised inequality and exploitation, foregrounding, inter alia, its racialised dimensions as an unfolding trauma where unvaccinated populations in the Global South are slated for disposability (Mbembe, 2019; on Covid-19 and racialised disposability).

Strikingly, despite the spectacular and divisive ways in which broader vaccine politics has played out in complex media publics, these critical notions have been widely disseminated. In response, a language of the commons has become widely deployed in public discourse to call not just for equal access to the vaccine as resource, but to stress the political imagination of interdependence and solidarity rendered fundamental by planetary virality. An appeal led by Nobel laureates has called for Covid-19 vaccines to be declared a ‘global common good’; a coalition of development and social justice non-governmental organisations has established the People's Vaccine Alliance to challenge the regime of patents and proprietary licensing which prevents the scaled-up and distributed production of generic vaccines. Grassroots protests across continents have garnered significant coverage in pressing for equitable vaccine access (Young, 2021). Even an ostensibly charitable mobilisation, UNICEF's ‘Get a Vaccine, Give a Vaccine’ campaign, draws centrally on an imagination of irreducible global interdependence. In the campaign video, the actor Liam Neeson repeats the campaign's central message – ‘Wealthy countries are racing to vaccinate their populations yet billions of people in poor countries don't have any vaccines. Scientists tell us that no one is safe until everyone is safe.’

Vaccine inequality, therefore, and the systemic conditions which produce and sustain it, has emerged as the clearest pandemic focal point for a commitment to commoning. It has arguably done so in a register that succeeds in communicating the implacable dimensions of a globalised condition. While opinion polls provide for a very limited engagement with the complexities of ‘public opinion’, national surveys in the UK and USA, for example, have consistently found popular support for increasing vaccine donation by national governments (Mori, 2021; Silverman, 2021). A major international study published in *Nature Medicine*, found, to quote ‘a remarkable uniformity of opinion across countries with between 48% and 56% supporting some level of donation of vaccine stockpiles. Of those that supported vaccine donations, over 70% favoured donating at least 10% of their country's doses’ (Clarke et al., 2021).

Returning to Gross's discussion of ‘scripts of hope’, it is plausible to suggest that vaccine justice has achieved a notable legibility, rendering transparent injustice and its consequences, and articulating solutions based on varying intensities of commoning (and, impressionistically at least, it has done so in the face of far more sustained media interest in the spectacle of ‘anti-vaxx’ politics). Nevertheless, the clearest mobilisation around a politics of commoning runs up against predictable and implacable problems of political agency at scale. Humanitarian drives are dependent on the intensity of publicity cycles that certainly provide short-term results but that also contribute, when this intensity wanes, to a sense that this issue is ‘solved’. Governmental, party political and civil society actions aimed at legal and institutional solutions, such as for example the calls to implement a TRIPS waiver,^1^ are mired in the process of lobbying at international level, where the EU, for example, remains committed to refusing any such waiver, citing the protection of key industries and foregrounding charitable initiatives as a counter to political ‘shame’. More radical solutions, that call, for example, for large pharmaceutical companies to be taken under democratic control to facilitate the emergency expansion of vaccine production and distribution, have little more than rhetorical power behind them. At the time of writing, it is the politics of competitive advantage which have been given a shot in the arm: wealthy nations have largely opted for a policy of issuing ‘booster vaccines’, despite repeated calls from the World Health Organization to place a moratorium on this approach until every country globally has been able to vaccinate at least 40% of the population (Keaten, 2021).

## Publicness with contingent commoning

As noted in the Introduction, the disjunctive effects of the pandemic are widely held to have rendered systemic problems transparent in the sense of being undeniable, forging a potential basis for thinking about forms of remedy and transformation. The pandemic has thus been widely hailed as a *crisis*, where the idea of crisis encompasses both a material reality – of epidemiological relations, and thus of the institutions, services and social relations which must respond to this reality – and a discursive one, where narratives of causality compete to explain and frame its nature, extent and significance. For progressive and radical politics, focused on what follows the rendering transparent of the pandemic's inequalities and injustices, the disjuncture has been broadly conceived of as a critical turning point which can sustain a refusal of returning to ‘business as usual’.

This brief discussion highlights the resurgence in forms of large-scale transformative speculation addressing the pandemic from within, imagining the ‘world after’ not only in terms of the impacts and consequences for potential futures, but also of the kinds of dispositions, collectives and generative antagonisms which may take deeper or emergent form. If the previous analysis hinged on commoning as an end goal (vaccine justice), this more speculative intellectual activity addresses how the pandemic may drive commoning as a process of cooperation and solidarity.

Slavoj Žižek, for example, argues that the pandemic challenges us to accept both that a ‘new way of life will have to be invented’ but that this will not emerge from imagining its ‘end’ – rather it must be conceived of as ‘announcing a new era of ecological troubles’ (Žižek, 2021: 9). For Bruno Latour, similarly, the pandemic must be understood as folded into the ecological crisis. Reflecting on the disorienting conditions of confinement as simultaneously involving paralysis and mobilisation, Latour argues that the abrupt, systemic disruption of global economic activity has unsettled ‘the economy’ as an ‘unsurpassable horizon’ of human activity, providing us with the possibility to address the disconnection between ‘la monde où l’on vit’ (the world where we live, as located consumers, citizens, subjects, the symbolic world that informs us) and ‘la monde dont on vit’ (the world we live in and which sustains us, materially and ecologically) (Latour, 2021).

In a comparable reflection on what follows a stark renewal of recognition of relations of dependence and interdependence, Benjamin Bratton stakes out the advent of an ‘epidemological view of society’, that is, the disruption of contractual and atomised visions of social relations to quotidian ways of seeing society as an interdependent biological community, thus shifting ‘our sense of subjectivity away from private individuation and towards public transmissibility’ (Bratton, 2021: loc. 393). This forces us to rethink basic dynamics between individual and society. Contra liberal and neoliberal imaginaries of societies comprised of self-governing individuals and sovereign consumers, the pandemic demands that we see ourselves as implicated in processes of transmission, and subject to forms of risk that cannot easily be individuated:

The pandemic has made it easier to see oneself more as a node in a biopolitical network to which one is responsible than as an autonomous individual whose sovereignty is guaranteed by free will or in the image of the national autocrat's symbolic prestige, at least for most people. (Bratton, 2021: loc. 405)

As with Latour, Bratton discerns a shift in patterns of identification and symbolic interaction which can be secured to and developed as a basis for forms of commoning. But this shift is, as he recognises, ambivalent, ‘for some an affront to identity and for others its precondition’ (Bratton, 2021: loc. 405). The duality of crisis may generate forms of identification and imagination commensurate with its material and biological complexity, but it also re-animates forms of displacement and denial under conditions of complex publicness. As several commentaries have also explored, the realisation of profound rupture, in the absence of ‘feasible political alternatives’, promotes feelings of being ‘locked in’. This can further drive forms of *rejection* – not only of being subject to governance and expertise, but also the horizontal relations of interdependence and solidarity necessary to processes of commoning, as evidenced by the consistent externalising fantasies of anti-immigration politics (see Opratko et al., 2021). Commenting on the circulation of conspiracies focused on the very quotidian shift in epidemiological relations considered by Bratton – mask-wearing, vaccine uptake – Nicolas Guilhot approaches them less as cognitive mis-steps than as failures of persuasive visions of a ‘common world and inclusive future’:

Never before has our existence as individuals and as a species felt so precarious. Never has our world seemed so fragile. Our capacity to project ourselves in the future has shrunk dramatically … yet it is also business as usual. One looks in vain for the cultural and political resources that would help us see through the apocalyptic haze the possibility of a new beginning, and a better one.  (Guilhot, 2021)

The implacable biological reality of the pandemic has informed significant attempts to speculate as to how the stark awareness of relations of interdependence can be compounded and directed towards a desire for more generative social relations and life-in-common. Yet this publicness with contingent commoning must reckon with the ways in which, inter alia, nationalism and nihilistic politics responds to the same set of realisations with a spectacular investment in imaginaries of exclusion and abasement.

## Commoning without publicness

What does the project of publicness look like from the social margins, and in period of intense mediation of a protracted crisis? What actors and narratives are absent from the early draft of the Covid-19 pandemic's history? Widely circulated images of ‘the masses’ of presumably infectious urban poor, or stories of minorities breaking lockdown rules have been making the news rounds across many contexts. Those in the periphery of urban, national and global geographies often appear as no more than corporeal excess in conditions of high risk. As the media direct audiences’ gaze outwards and downwards, with the state and its corporate partners reaffirmed as the core authority to manage – non-human and human – risk, publicness at the social margin is rendered as yet another threat.

Within the political imagination of crisis, those already marginalised by the market and the state are caught within a paradoxical space of publicness: regularly seen as embodying risk but remaining unrecognised as subjects whose lives matter. Even before the pandemic, the public lives of racialised and classed minorities were intensely policed, with young Black men in London 19 times more likely to be stopped and searched (Dodd, 2020b), a discrimination intensified during lockdowns, with police twice as likely to fine black people over Covid-19 rule breaches (Dodd, 2020a). Socio-technical assemblages of bordering (Chouliaraki and Georgiou, in press), in the form of surveilling state mechanisms and sensationalist media narratives, reproduced the familiar binary of victim/perpetrator for those at the margins. The urban working class was swiftly contained in a binary frame of selective publicness: a frame that allowed classed and racialised frontline workers sympathy and permission to go out to work, but withheld consent and recognition for those whose public appearance seemed unjustified on the basis of health and economic need.

Against permitted and institutionalised conceptions of publicness, horizontal networks of connection and solidarity in urban neighbourhoods rejected the binary of victim/perpetrator, instead engaging in a local and affective politics of care. Care, according to Ahmed (2017), is a kind of political warfare in conditions of precarity, or as Ticktin (2020) puts it, ‘to engage in care is to uphold the right to survive’. The hundreds of mutual aid groups, initiated digitally and enacted in practices and communication of solidarity on and offline across London's neighbourhoods, represent a striking example of commoning, even if ephemeral (or perhaps precisely because of this ephemerality). Mobilising the value of mutuality and horizontality of localised care, these networks shifted urban publicness away from conceptions of threat to publicness as radical hope (Gross, 2021). Importantly, the groups were built on the basis of mutuality and interdependence, recognising the value of life and wellbeing to be protected not on the basis of pre-existing relations but on the basis of the collective threat. Deliberately organised across networks – on social media but also through analogue and phone communication for those not on social media – the mutual aid groups opened up spaces for reimagining socially and racially fragmented neighbourhoods by offering unconditional mutual support, engaging in this way in ‘the production and reproduction of ourselves as a common subject’ (Ticktin, 2020).

Mutual aid groups became sites of possibility for the realisation of a *networked commons*: digitally enabled and contingent associations through which people may generate meaningful community relations outside the remit of the state or the private sector (de Peuter and Dyer-Witheford, 2010) and their production of ‘precarious labour, precarious stay and precarious lives’ (Trimikliniotis et al., 2015: 1). At the heart of this generative process of commoning lies the experience of ‘throwntogetherness’ (Massey, 2005); the dynamic potentiality of the convergence of different histories, experiences and actions, posing ‘that most fundamental of political questions … how are we going to live together’ (Massey, 2005: 15). This political understanding of publicness, which brings together discourse, practice and affect, illustrates how public spaces of marginality can emerge as ephemeral, even contradictory, sites of contestation, generating solidarity and togetherness against media's divisive discourse and the state's punitive mechanisms of surveillance.

In the context of the pandemic and its regulated publicness, commons emerged in active processes of ephemeral connections and collective claims – as commoning. The short-lived commitment of the mutual aid groups to politics of solidarity and care is both a powerful example of commoning and of its limits. It suggests the limits of crisis-driven connection and action on the one hand, but also the desire for collective responses to precarity, isolation and individualised solutions to a systemic crisis. Commoning proved that, even when not driven by predetermined answers and pre-existing collectivities, it can generate collective and radical responses to crisis.

## Conclusion

This analysis is concerned with the ways in which publicness and commoning have intersected during the Covid-19 global pandemic. The acceleration of the pandemic rendered starkly both biological and systemic interdependences and socio-economic inequalities. Its disjunctive effects have informed a palpable desire to ensure that its unsettling synchronicities and manifest injustices infuse collective desires for something better: a transformation in human impacts on the ecologies that sustain human life, profound changes in the socio-economic systems which devalue and destroy many lives and etch classed, gendered and racialised lines in human societies. The idea of the commons, in its most capacious sense, captures this desire for transformed resources and transformative relations. This argument traces three ways in which visions of commoning have been articulated during the pandemic, under conditions of complex publicness. Hope for change is widely based on the conviction that the pandemic has exposed unequal and unsustainable conditions in ways that can no longer be denied, an investment in public revelation that must reckon, at every turn, with the dynamics of complex publicness.

As the three heuristic discussions suggest, the intersection of publicness and commoning is contingent in ways that resist prescription, and the value and character of publicness diverges radically according to scale and relation – maximum publicity is a necessary condition for asserting a vaccine commoning in the face of racialised inequalities in the international system, yet it is a threat to those whose intensified social and political marginalisation, in this period, deepens the racialised inequalities within the nation-state. And yet, our insistence on the ambivalent effects of complex publicness is not to deny the unsettling force of the pandemic. Rather, it draws attention to the value of horizons and projects of commoning as endeavours that recognise that they are always necessarily incomplete mobilisations aimed at social and political reconstitution (Harvey, 2011).

Asking what common world can be imagined, and what common action is possible, we have attempted to explore the potentialities of the current disjuncture for ‘scripts of hope’. Visions and practices of commoning confront structural containment, assertive nationalisms and the brutal marketisation of life-saving medication and cooperation. Concomitantly, they re-instate critical questions of justice and humanity at global, national and localised scales, and through heterogenous and diverse connectivities, digital and embodied, they refuse a return to the ‘old normal’ or the construction of a ‘new’ but similarly unequal ‘normal’.

## Notes

1. The TRIPS agreement is the Agreement on Trade-Related Aspects of Intellectual Property Rights, binding members of the World Trade Organization (WTO). The proposal of a TRIPS waiver for Covid-19 medical resources would remove the legal barriers presented by patents and licences to the states of and producers in ‘low- and middle-income countries’ in manufacturing vaccine supplies.
